# Silver Nanoparticles and Polyphenol Inclusion Compounds Composites for *Phytophthora cinnamomi* Mycelial Growth Inhibition

**DOI:** 10.3390/antibiotics7030076

**Published:** 2018-08-16

**Authors:** Petruta Mihaela Matei, Jesús Martín-Gil, Beatrice Michaela Iacomi, Eduardo Pérez-Lebeña, María Teresa Barrio-Arredondo, Pablo Martín-Ramos

**Affiliations:** 1 Department of Bioengineering of Horticultural and Viticultural Systems, University of Agricultural Sciences and Veterinary Medicine of Bucharest, Bulevardul Mărăști 59, București 011464, Romania; petruta.matei@horticultura-bucuresti.ro (P.M.M.); b.iacomi@yahoo.fr (B.M.I.); 2 Agriculture and Forestry Engineering Department, ETSIIAA, Universidad de Valladolid, Avenida de Madrid 44, 34004 Palencia, Spain; mgil@iaf.uva.es (J.M.-G.); eplebena@gmail.com (E.P.-L.); 3 Centro de Salud Barrio España, Sanidad de Castilla y León (SACYL), Calle de la Costa Brava, 4, 47010 Valladolid, Spain; jesusmartingil@gmail.com; 4 Department of Agricultural and Environmental Sciences, EPS, Instituto de Investigación en Ciencias Ambientales (IUCA), University of Zaragoza, Carretera de Cuarte, s/n, 22071 Huesca, Spain

**Keywords:** antifungal, chitosan oligomers, composites, deep eutectic solvents, phenolic compounds, *Phytophthora cinnamomi*, root rot, silver nanoparticles

## Abstract

*Phytophthora cinnamomi*, responsible for “root rot” or “dieback” plant disease, causes a significant amount of economic and environmental impact. In this work, the fungicide action of nanocomposites based on silver nanoparticles and polyphenol inclusion compounds, which feature enhanced bioavailability and water solubility, was assayed for the control of this soil-borne water mold. Inclusion compounds were prepared by an aqueous two-phase system separation method through extraction, either in an hydroalcoholic solution with chitosan oligomers (COS) or in a choline chloride:urea:glycerol deep eutectic solvent (DES). The new inclusion compounds were synthesized from stevioside and various polyphenols (gallic acid, silymarin, ferulic acid and curcumin), in a [6:1] ratio in the COS medium and in a [3:1] ratio in the DES medium, respectively. Their in vitro response against *Phytophthora cinnamomi* isolate MYC43 (at concentrations of 125, 250 and 500 µg·mL^−1^) was tested, which found a significant mycelial growth inhibition, particularly high for the composites prepared using DES. Therefore, these nanocomposites hold promise as an alternative to fosetyl-Al and metalaxyl conventional systemic fungicides.

## 1. Introduction

Nanotechnology has shown remarkable applications in biomedicine, diagnosis and antibacterial treatments, and is now transforming the agricultural sector, particularly with the development of novel nanopesticides and nanofertilizers [1]. The increase in the frequency of resistant or tolerant pathogenic agents, which has in turn led to an excessive application of pesticides, has resulted in an increase in the presence of residues in food products, which may pose a major risk to health. The design and testing of safe, effective and environmentally sustainable formulations based on nanoemulsions, nanocomposites and nanoparticles to control agricultural pests and pathogens has become a burgeoning field of research in the past few years.

Silver, which has long been used as a disinfectant for pathogenic microorganisms [2], has become one of the best exponents of this transition. Silver nanoparticles (AgNPs), which feature antibacterial, antifungal and antitumor activities [3,4,5], are one of the most popular active ingredients employed to enhance the efficacy of plant protection products. Furthermore, they can be prepared through green synthesis procedures with the aid of plant extracts [6], which act as reducing and stabilizing agents. Polyol components, polysaccharides, and water-soluble heterocycles (such as those from *Stevia rebaudiana* [7], *Curcuma longa* [8], *Pongamia pinnata* [9], *Gliricidia sepium* [10], *Eucalyptus hybrida* [11], or *Quercus brantii* [12]) have been reported to lead to a synergistic effect in the resulting phytonanocomposites [13,14,15].

Nonetheless, bioactive compounds from plants (that include phenolic acids, flavonoids, curcuminoids, coumarins, quinones, tannins and lignans), in spite of having a wide range of activities, generally suffer from a number of drawbacks derived from their inherent physicochemical characteristics (poor water solubility, low bioavailability, chemical instability, photodegradation, rapid metabolism and short half-life) [16], which limit their applications. To stabilize them and improve their bioavailability, one well-known approach is to use biopolymers, such as chitin, chitosan, starch, and cellulose, or other macromolecular systems [17]. Binary composites based on chitosan with polyphenols (e.g., gallic acid or curcumin [18,19,20]) and ternary composites that also include AgNPs [21,22] with a broadened bio-activity have been reported in the literature. 

Other approaches to improve solubility are based on forming inclusion compounds with terpene glycosides (such as rubusoside, stevioside, rebaudioside, or steviol monoside) or cyclodextrins, which result in an enhancement of the solubility of polyphenols [23,24], or on using deep eutectic solvents (DES). DES are an excellent extraction medium for phenolic compounds [25] and may be used in combination with inclusion compounds [26] or with chitosan [27,28].

*Phytophthora cinnamomi* is a pathogen with over 1000 host species, transmitted by the soil and which causes rotting of the roots of many horticultural and forestry crops [29]. *P. cinnamomi* can collapse, which cause sudden death of plants and a decrease in fruit yield and size. The infection by *P. cinnamomi* can also occur together with other species of *Phytophthora*, mainly *P. cambivora*, *P. cryptogea*, *P. citricola* and *P. cactorum*. Its eradication by means of fungicides is expensive and causes damage to the environment, and fumigation is not always effective for deeper roots [30,31]. Consequently, the European Union is promoting the development of new natural bioactive compounds to replace conventional systemic fungicides, such as the organophosphorus compound fosetyl-Al or acylalanines such as metalaxyl.

It has been shown, in vitro, that AgNPs synthesized using aqueous plant extracts have had antifungal effect on *Phytophthora* pathogens [32], and so do chitosan [33], the binary combinations of the two [34] and their ternary combinations with propolis [35]. Nonetheless, to the best of the authors’ knowledge, no studies have explored the use of composites of AgNPs with polyphenol inclusion compounds, combined either with chitosan oligomers (COS) or with DES, for the control of *P. cinnamomi* or any other oomycetes.

In the present study, four polyphenols (gallic acid, silymarin, ferulic acid and curcumin) were assessed for the microwave-assisted formation of the new inclusion compounds with stevioside. AgNPs were subsequently incorporated into the composites [36]. Two types of host matrices were tested, namely COS in a hydroalcoholic solution, and a DES based on a choline chloride and urea solution (1:2 *v/v*) in glycerol, evaluating in vitro their response against *P. cinnamomi* at different concentrations.

## 2. Results

**Figure 1:**
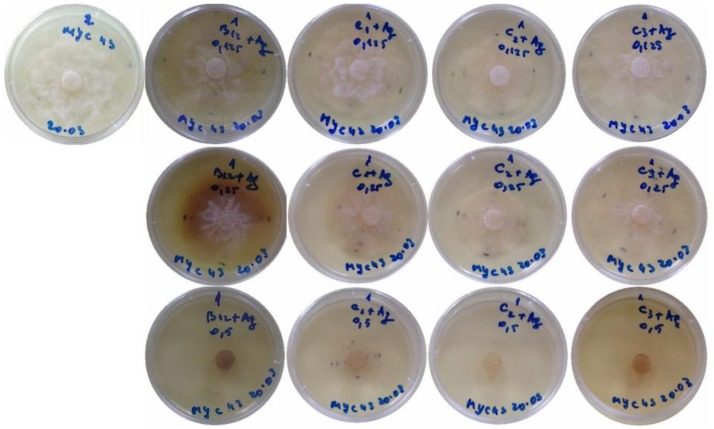
*Phytophthora cinnamomi* growth inhibition assays with the nanocomposites based on the chitosan oligomers in hydroalcoholic solution preparation medium. *From left to right*: Control (no treatment) and treatments with AgNPs combined with gallic acid, silymarin, ferulic acid and curcumin inclusion compounds. *From top to bottom*: 125 µg·mL^−1^, 250 µg·mL^−1^, and 500 µg·mL^−1^ concentrations. Only one repetition per treatment is shown.

**Figure 2:**
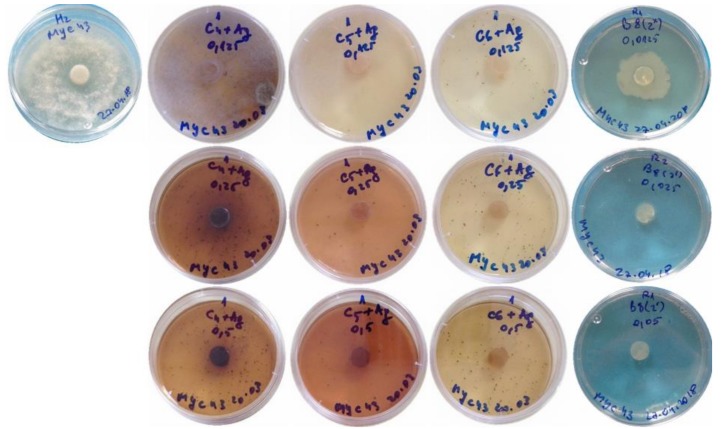
*P. cinnamomi* growth inhibition assays with the nanocomposites based on the deep eutectic solvent preparation medium. *From left to right*: Control (no treatment) and treatments with AgNPs combined with gallic acid, silymarin, ferulic acid and curcumin inclusion compounds. *From top to bottom*: 125 µg·mL^−1^, 250 µg·mL^−1^ and 500 µg·mL^−1^ concentrations. Only one repetition per treatment is shown. The blue background in the control and curcumin-treated samples is due to the blue color of the paper on which the plates were on.

The antifungal activity of the different treatments in aforementioned two preparation media (COS in hydroalcoholic solution and DES) against *P. cinnamomi* (isolate MYC43) was studied in vitro by monitoring the radial growth of the mycelium (Figure 1 and Figure 2).

**Figure 3:**
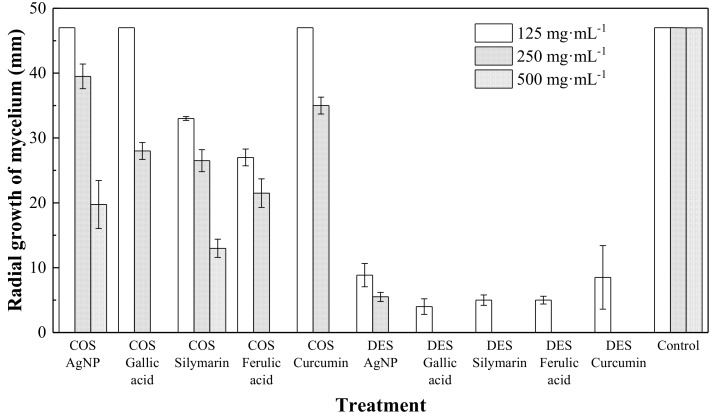
Radial growth values of *P. cinnamomi* in the presence of the composites, which consisted of different polyphenol inclusion compounds and silver nanoparticles at different concentrations, either in a chitosan hydroalcoholic solution (COS) or in a deep eutectic solvent (DES). A control (no treatment) and two treatments with AgNPs (in COS and DES) without the inclusion compounds are shown for comparison purposes. Error bars represent the standard deviation across three replicates.

As shown in Figure 3, the increase in the concentration of the inclusion complexes from 125 µg·mL^−1^ to 500 µg·mL^−1^ resulted in a reduction in the radial growth of the mycelium in all cases. It may be observed that 100% mycelial growth inhibition occurred with the COS medium at the highest concentration of 500 µg·mL^−1^ for the composites with gallic acid, ferulic acid and curcumin (but not for silymarin). On the other hand, at lower concentrations (125 and 250 µg·mL^−1^), silymarin and ferulic acid-based treatments were more effective than those based on gallic acid and curcumin.

As regards the nanocomposites with a DES preparation medium, total inhibitory activities were attained for the composites based on the four polyphenols under study concentrations of 250, and 500 µg·mL^−1^. Further, at the lowest concentration of 125 µg·mL^−1^, the antifungal performance of the composites in the DES medium was close to 90% for all polyphenols. Thus, the product efficacies were clearly higher than those of the composites based on the hydroalcoholic solution of COS.

Upon comparison with the treatments without phenolic inclusion compounds, it could be observed that the AgNPs-only treatment attained a lower inhibition than the composites for the COS medium, with mycelium growth even at the highest dose. On the other hand, the DES-based AgNPs-only treatment performed noticeably better than its COS counterpart, albeit it did not completely inhibit growth at a concentration of 250 mg·mL^−1^ (whereas all the composites did). Therefore, an enhanced fungal growth control activity of the ternary mixtures was evidenced.

The results from the sensitivity tests for *P. cinnamomi* may also be expressed with the help of EC_50_ and EC_90_ indicators (Table 1). The sensitivity of the isolate mainly varied as a function of the preparation media, but also according to the phenolic compound used. In line with the discussion presented above, *P. cinnamomi* (MYC43) was found to be remarkably more sensitive to the treatments prepared in DES, with EC_50_ values ranging from 0.1 to 8.9 µg·mL^−1^ and EC_90_ values between 77.9 and 184.3 µg·mL^−1^. For comparison purposes, for the COS in hydroalcoholic solution medium treatments the EC_50_ values ranged from 171.6 to 279.9 µg·mL^−1^ and those of EC_90_ from 450.4 to 963.7 µg·mL^−1^.

The highest sensitivity of the *P. cinnamomi* isolate (MYC43) corresponded to the inclusion compound with gallic acid in DES (EC_50_ = 0.1 µg·mL^−1^), followed by the inclusion compounds with silymarin and ferulic acid, and finally by the one with curcumin, with EC_50_ values of 0.6, 0.6 and 8.9 µg·mL^−1^, respectively.

## 3. Discussion

With a view to comparing the efficacy of the proposed nanocomposites verses other phenolic-based products against *Phytophthora* spp. discussed in the literature, it should be noted that Kim et al. [37] reported strong fungicidal activities of *Curcuma longa* L. rhizome-derived curcumin in ethyl acetate and hexane fractions against *P. infestans* with 100% and 84% control values at a concentration of 1000 µg·mL^−1^. Apart from the higher concentrations used in those experiments as compared to the ones presented herein, it should be noted that a contribution of the pronounced cytotoxic activities of the solvents could not be excluded.

Pompimon et al. [38] assessed the anti-*P. capsici* activity of *C. longa* in acetone fraction, finding an inhibition of mycelial growth of ca. 90% at a concentration of 300 µg·mL^−1^, higher than the EC_90_ of 184 µg·mL^−1^ of the curcumin-based treatment in DES media reported in this study.

The nanocomposites of the four polyphenols in DES medium would also be more active than, for instance, cuminic acid, which featured an EC_50_ value against mycelial growth of *P. capsici* of 19.7 µg·mL^−1^ (which in turn was lower than the EC_50_ value of other benzoic acid derivatives in previous reports) [39]. Other natural compounds, such as furanocoumarins (e.g., psoralen or isopsoralen) would require concentrations of 500 µg·mL^−1^ to attain 82–84% disease control against *P. infestans* [40].

An EC_50_ value of amphopolycarboxyglycinate-stabilized AgNPs against *P. infestans* of 3.1 µg·mL^−1^ was reported by Krutyakov et al. [41], i.e., a 30 and 6 times higher concentration than those obtained for the AgNPs combined with gallic acid, silymarin and ferulic acid inclusion compounds in DES, respectively.

Banik and Pérez-de-Luque [42] found that the integration of copper nanoparticles (CuNPs) with non-nano copper like copper oxychloride, both at a 50 µg·mL^−1^ concentration, resulted in a 76% growth inhibition in vitro of the oomycete *P. cinnamomi* as compared to the control. Since in comparative assays between NPs the concentrations of AgNPs are usually 10 times higher than those CuNPs [43,44], the equivalent concentration of AgNPs to attain aforementioned effects should be 500 ca. µg·mL^−1^, four times higher than that required by the DES treatments to attain a comparable mycelial growth inhibition (ca. 90%).

Chitosan has also been assayed against *P. infestans* [33], finding that concentrations of 500 µg·mL^−1^ would be required to fully inhibit mycelial growth, similar to those of the COS-based nanocomposites with gallic acid, ferulic acid and curcumin in this study. On the other hand, *N*-(6-carboxyl cyclohex-3-ene carbonyl) chitosan with different degrees of substitution achieved an EC_50_ of 298 µg·mL^−1^ for *P. infestans* [45], better than the ones for the COS composites based on the three aforementioned polyphenols (in the 450–490 µg·mL^−1^ range).

The overall efficacy of the reported nanocomposites should be referred to the combination of the properties afforded by each of its constituents and their synergies.

According to Kim et al. [46], nanosilver may exert an antifungal activity by disrupting the structure of the cell membrane and inhibiting the normal budding process due to the destruction of the membrane integrity. Silver nanoparticles antifungal action may also result from the release of silver ions into the intracellular matrix of the pathogen [47]. Reports on the mechanism of inhibitory action of silver ions on microorganisms have shown that upon treatment with Ag^+^, DNA loses its ability to replicate, resulting in inactivated expression of ribosomal subunit proteins, as well as certain other cellular proteins and enzymes essential to ATP production. It has also been hypothesized that Ag^+^ would affect the function of membrane-bound enzymes, such as those in the respiratory chain [4].

Apropos of the role of the stevioside, the improvement in the solubility and bioavailability of the polyphenolic compounds should be ascribed to the formation of a nanocomposite structure comprising a transglycosylated compound, which includes the insoluble compounds. Transglycosylated materials have been reported to self-associate into particular micelle-like structures with a core-shell-like architecture, in which the hydrophobic skeleton is segregated from the aqueous exterior to form a novel drug-loading core, surrounded by a hydrophilic shell of sugar groups [48]. For instance, Kadota, Okamoto, Sato, Onoue, Otsu and Tozuka [24] found a 13000× increase in curcumin solubility when the tri-component system curcumin/*α*-glucosyl stevia/polyvinylpyrrolidone was used.

In relation to the phenolic compounds, they have been reported to have toxic activities against fungi involved in the deterioration of agricultural products by interfering with the development of mycelia [49]. They affect membrane functions such as electron transport, nutrition, enzyme activity, protein and nucleic acid synthesis, and they interact with membrane proteins, causing disruption of the structures and functionality. For instance, curcumin’s efficacy would be influenced by its lipophilic nature, which leads to an adequate transmembrane permeability [16]. Its antifungal mechanism has been ascribed to the disruption of plasma membrane integrity, causing leakage of potassium ion from the cytosol and change in membrane potential [50]. On the other hand, gallic acid would exhibit both antioxidant as well as pro-oxidant characteristics, displaying a dual-edge sword behavior, which turns it into an efficient apoptosis inducing agent [51].

Regarding the inhibition mode of chitosan, three mechanisms have been proposed [52]: (1) Its positive charge can interact with negatively charged phospholipid components of fungi membrane, increasing its permeability and causing the leakage of cellular contents, which subsequently leads to cell death; (2) it can act as a chelating agent by binding to trace elements, causing the essential nutrients unavailable for normal growth of fungi; and (3) it may be able to penetrate the cell wall of fungi and bind to its DNA, inhibiting the synthesis of mRNA and, thus, affecting the production of essential proteins and enzymes.

As far as DES are concerned, they would act as a plasticizer, affecting the apparent viscosity of the solutions and enhancing water vapor permeability, water solubility and water sorption capability, as reported by Almeida, Magalhães, Souza and Gonçalves [27]. Nonetheless, it worth noting that, while choline chloride and urea show no inhibition as individual materials, their final product as ChCl:U DES has been reported to show inhibition towards *Candida cylindracea* [53]. This behavior could be due to the synergistic effect of forming DES [54] and can be used to prove that occasionally DES have a higher toxicological behavior than its original components.

The antimicrobial activity of DES is still not fully understood [55]. Some reports have noted that DES would increase the permeability of the lipid membrane of eukaryotic cells [56,57], as chitosan does. Since the mechanism for COS and DES would tentatively be similar, the differences in the performance of the composites presented herein in terms of fungal growth control should then be ascribed to differences in their ability to solubilize a wide range of solutes (e.g., components in the fungal cell membrane), pH, osmolality or chelation of membrane-bound divalent cations [58].

Since one of the possible mechanisms of action of silver requires that the silver ions enter the fungal cell for efficient killing, the enhancement of permeability driven by COS, DES and polyphenols would support that their interaction should be synergistic rather than simply additive.

From our work, the best results of mycelial growth inhibition at the lowest concentration (125 mg·mL^−1^) in DES (GI 91.5%), attained for the composite based on gallic acid, may be ascribed to the fact that gallic acid is extremely well absorbed, and very soluble in water as compared with other polyphenols [51]. Moreover, the introduction of the hydroxyl group on the cation in the chloride of choline salt has also been reported to significantly improve the extraction capacity of ionic liquids for gallic acid [59].

## 4. Materials and Methods

### 4.1. Reagents

Stevioside standard was purchased from Wako (Osaka, Japan). Gallic acid, silymarin, ferulic acid, curcumin, choline chloride, urea, glycerol, and silver nanoparticles (40 nm particle size (TEM), 0.02 mg·mL^−1^ in aqueous buffer, with sodium citrate as a stabilizer) were purchased from Sigma-Aldrich/Merck KGaA (Darmstadt, Germany).

Chitosan oligomers were obtained from medium molecular weight chitosan (supplied by Hangzhou Simit Chemical Technology Co., Ltd., Hangzhou, China), dissolving 10 g in 500 mL of acetic acid (1%) under constant stirring at 60 °C. Once dissolved, hydrogen peroxide (0.3 mol·L^−1^) was added for the degradation of the polymer chains, obtaining oligomers of less than 2000 Da [60].

Liquefaction of the choline chloride and urea (1:2 *v/v*) eutectic mixture occurred at 80 °C under stirring in a hot-plate magnetic stirrer for 10 min, in good agreement with Biswas et al. [61].

### 4.2. Microwave-Assisted Preparation of the Polyphenol Inclusion Compounds

The aqueous biphasic system separation technique was used for the formation of the inclusion compounds. This technology is a liquid-liquid extraction system for bioseparation and is frequently used to process all types of biotechnological materials, such as proteins, enzymes, phytochemicals, nucleic acids and pigments [62]. In the case under study, the inclusion complexes formed with stevioside were recovered from the upper part of the reactor.

### 4.3. Chitosan Oligomers (COS)-Based Composites

In each of the four jars with screw caps, 1 g of chitosan oligomers of 2000 Da (COS), 0.06 g of stevioside and 0.01 g of one of the polyphenols (either gallic acid, silymarin, ferulic acid or curcumin) were added to 40 mL of hydroalcoholic solution (1:1 *v/v* distilled water and ethanol), followed by treatment in a microwave (Milestone Ethos-One, Sorisole, BG, Italy) at 80 °C for 20 min under stirring. The resulting solutions were centrifuged at 2500 rpm and stored at 4 °C. For the incorporation of the silver nanoparticles to the nanocomposite, 0.1 mL of commercial AgNPs (0.02 mg·mL^−1^) were added dropwise to 0.9 mL of the previously centrifuged microwave fractions, and the final solution (with pH 7.5) was stirred at room temperature.

### 4.4. Deep Eutectic Solvent-Based Composites

To prepare the DES-based composites, 20 mL of choline chloride and urea (1:2 *v/v*) DES and 10 mL of glycerol were added to each of the four jars with screw caps, together with 0.03 g of stevioside and 0.01 g of the respective polyphenol (gallic acid, silymarin, ferulic acid or curcumin). The mixture was heated at 80 °C in the microwave, under stirring, for 20 min. Then, 0.1 mL of AgNPs (0.02 mg·mL^−1^) were added dropwise to 0.9 mL of the microwave fractions (previously centrifuged at 2500 rpm). The mixture, with pH 7.5, was subjected to vigorous stirring for 5 min at room temperature.

### 4.5. Silver Nanoparticles-Only Treatments

Two additional treatments consisting of 0.1 mL of AgNPs (0.02 mg·mL^−1^) added dropwise to 0.9 mL of the dispersion medium (either COS or DES), without the polyphenol inclusion compounds, were prepared for control purposes. The mixtures were vigorously stirred for 5 min at room temperature.

### 4.6. Characterization of the Nanocomposites

The structure and properties of the nanocomposites obtained through the synthetic procedures described above were characterized using Fourier-Transform Infrared spectroscopy (FTIR), scanning electron microscopy (SEM) and transmission electron microscopy (TEM). Results were recently reported in patent P201731489 [36].

### 4.7. Fungal Isolates and Growth Conditions

The fungal species used in the experiment was *Phytophthora cinnamomi* isolate MYC43, supplied by Centro de Investigaciones Científicas y Tecnológicas de Extremadura—Instituto del Corcho, la Madera y el Carbón Vegetal, Spain. The isolate was maintained in potato-dextrose-agar (PDA) slant tubes, supplied by Merck Millipore (Darmstadt, Germany), stored at 4 °C.

### 4.8. Efficacy of the Nanocomposites for the Control of Phytophthora cinnamomi

Agar disks (8 mm in diameter) were cut from the margin of a 7-day-old colony growing on PDA and were transferred to a PDA medium supplemented with the nanocomposites at final concentrations of 125 µg·mL^−1^, 250 µg·mL^−1^ and 500 µg·mL^−1^. Three replicates were performed for each treatment. For each active ingredient and concentration, inhibition of radial mycelial growth (mm) compared with the untreated control was evaluated after 7 days of incubation at 24 °C, in the dark. The relative growth inhibition (%) of each treatment compared to untreated control was calculated as follows: Growth inhibition (%) = [(*dc* − *dt*)/*dc*] × 100, where *dc* stands for the average diameter of the fungal colony in the control and *dt* is the average diameter of the treated colony [63]. Results were expressed as effective concentrations EC_50_ and EC_90_ (i.e., the concentrations which reduced growth inhibition by 50% and 90%) by regressing the inhibition of radial mycelial growth values (% control) against the values of the antifungal nanocomposite concentrations.

## 5. Conclusions

Composites consisting of silver nanoparticles and polyphenol inclusion compounds were synthesized using two preparation media, one based on chitosan oligomers in an hydroalcoholic solution and the other based on a deep eutectic solvent. Both types of composites showed an increase in the water solubility of the polyphenols and in the in vitro antifungal activity against *Phytophthora cinnamomi* (MYC43 isolate). Nonetheless, the DES-based samples efficacy was remarkably higher than that of their counterparts with a chitosan oligomers-based matrix: Complete inhibition of mycelial growth was attained at concentrations of 250 and 500 µg·mL^−1^, and even at the lowest dose of 125 µg·mL^−1^, they resulted in a 90% growth inhibition. As regards the impact of the choice of the different polyphenols, for the DES-based treatments, the highest sensitivity of *P. cinnamomi* corresponded to the composite with gallic acid (EC_50_ = 0.1 µg·mL^−1^), followed by those with silymarin and ferulic acid, and finally by the one with curcumin, with EC_50_ values of 0.6, 0.6 and 8.9 µg·mL^−1^, respectively. This may be ascribed to the fact that gallic acid is extremely well absorbed, and very soluble in water as compared with other polyphenols. The reported activities for the composites were remarkably higher than those reported for *Phytophthora* spp. using AgNPs, chitosan or polyphenols separately. This points to the possibility of a successful application of these nanocomposites in agriculture, with the aim of reducing the use of toxic and expensive conventional systemic fungicides. Further research on the ability of the prepared nanocomposites to inhibit growth of *P. cinnamomi* in the context of a plant infection model and on the basic mechanism involved, fungistatic or fungicidal, is underway.

## 6. Patents

The work reported in this manuscript is related to Spanish patent P201731489.

## Figures and Tables

**Table 1:** Effective concentrations that inhibited mycelial growth by 50% and 90% (EC_50_ and EC_90_, respectively).

Treatment	EC_50_ (µg·mL^−1^)	EC_90_ (µg·mL^−1^)
COS AgNPs	458.4	1192.8
COS Gallic acid	261.3	455.6
COS Silymarin	261.8	963.7
COS Ferulic acid	171.6	450.4
COS Curcumin	279.9	487.4
DES AgNPs	13.3	253.3
DES Gallic acid	0.1	77.9
DES Silymarin	0.6	107.8
DES Ferulic acid	0.6	107.8
DES Curcumin	8.9	184.3

## References

[1] Kim D.-Y., Kadam A., Shinde S., Saratale R.G., Patra J., Ghodake G. (2018). Recent developments in nanotechnology transforming the agricultural sector: A transition replete with opportunities. J. Sci. Food Agric..

[2] Berger T.J., Spadaro J.A., Chapin S.E., Becker R.O. (1976). Electrically generated silver ions: Quantitative effects on bacterial and mammalian cells. Antimicrob. Agents Chemother..

[3] Xia Z.-K., Ma Q.-H., Li S.-Y., Zhang D.-Q., Cong L., Tian Y.-L., Yang R.-Y. (2016). The antifungal effect of silver nanoparticles on *Trichosporon asahii*. J. Microbiol. Immunol. Infect..

[4] Kim S.W., Jung J.H., Lamsal K., Kim Y.S., Min J.S., Lee Y.S. (2018). Antifungal effects of silver nanoparticles (AgNPs) against various plant pathogenic fungi. Mycobiology.

[5] Kharissova O.V., Dias H.V.R., Kharisov B.I., Pérez B.O., Jiménez Pérez V.M. (2013). The greener synthesis of nanoparticles. Trends Biotechnol..

[6] Rajan R., Chandran K., Harper S.L., Yun S.-I., Kalaichelvan P.T. (2015). Plant extract synthesized silver nanoparticles: An ongoing source of novel biocompatible materials. Ind. Crop. Prod..

[7] Bujak T., Nizioł-Łukaszewska Z., Gaweł-Bęben K., Seweryn A., Kucharek M., Rybczyńska-Tkaczyk K., Matysiak M. (2015). The application of different *Stevia rebaudiana* leaf extracts in the “green synthesis” of AgNPs. Green Chem. Lett. Rev..

[8] Sathishkumar M., Sneha K., Yun Y.-S. (2010). Immobilization of silver nanoparticles synthesized using *Curcuma longa* tuber powder and extract on cotton cloth for bactericidal activity. Bioresour. Technol..

[9] Raut R.W., Kolekar N.S., Lakkakula J.R., Mendhulkar V.D., Kashid S.B. (2010). Extracellular synthesis of silver nanoparticles using dried leaves of *Pongamia pinnata* (L.) Pierre. Nano Micro Lett..

[10] Raut W., Lakkakula R., Kolekar S., Mendhulkar D., Kashid B. (2009). Phytosynthesis of silver nanoparticle using *Gliricidia sepium* (Jacq.). Curr. Nanosci..

[11] Rahimi-Nasrabadi M., Pourmortazavi S.M., Shandiz S.A.S., Ahmadi F., Batooli H. (2014). Green synthesis of silver nanoparticles using *Eucalyptus leucoxylon* leaves extract and evaluating the antioxidant activities of extract. Nat. Prod. Res..

[12] Safaei H.R. (2017). Eco-friendly method for green recovery of silver nano particles from effluent fixer solution through interacting with *Q. brantii* (oak) peel hydro alcoholic extract. J. Adv. Eng. Technol..

[13] Li D., Liu Z., Yuan Y., Liu Y., Niu F. (2015). Green synthesis of gallic acid-coated silver nanoparticles with high antimicrobial activity and low cytotoxicity to normal cells. Process Biochem..

[14] Safarpoor M., Ghaedi M., Asfaram A., Yousefi-Nejad M., Javadian H., Zare Khafri H., Bagherinasab M. (2018). Ultrasound-assisted extraction of antimicrobial compounds from *Thymus daenensis* and *Silybum marianum*: Antimicrobial activity with and without the presence of natural silver nanoparticles. Ultrason. Sonochem..

[15] Alves T.F., Chaud M.V., Grotto D., Jozala A.F., Pandit R., Rai M., dos Santos C.A. (2017). Association of silver nanoparticles and curcumin solid dispersion: Antimicrobial and antioxidant properties. AAPS PharmSciTech.

[16] Hussain Z., Thu H.E., Amjad M.W., Hussain F., Ahmed T.A., Khan S. (2017). Exploring recent developments to improve antioxidant, anti-inflammatory and antimicrobial efficacy of curcumin: A review of new trends and future perspectives. Mater. Sci. Eng. C.

[17] Oliver S., Vittorio O., Cirillo G., Boyer C. (2016). Enhancing the therapeutic effects of polyphenols with macromolecules. Polym. Chem..

[18] Liu J., Pu H., Liu S., Kan J., Jin C. (2017). Synthesis, characterization, bioactivity and potential application of phenolic acid grafted chitosan: A review. Carbohydr. Polym..

[19] Hashemi Gahruie H., Niakousari M. (2017). Antioxidant, antimicrobial, cell viability and enzymatic inhibitory of antioxidant polymers as biological macromolecules. Int. J. Biol. Macromol..

[20] Saranya T.S., Rajan V.K., Biswas R., Jayakumar R., Sathianarayanan S. (2018). Synthesis, characterisation and biomedical applications of curcumin conjugated chitosan microspheres. Int. J. Biol. Macromol..

[21] Bajpai S.K., Ahuja S., Chand N., Bajpai M. (2017). Nano cellulose dispersed chitosan film with Ag NPs/curcumin: An in vivo study on albino rats for wound dressing. Int. J. Biol. Macromol..

[22] Barbinta-Patrascu M.E., Badea N., Pirvu C., Bacalum M., Ungureanu C., Nadejde P.L., Ion C., Rau I. (2016). Multifunctional soft hybrid bio-platforms based on nano-silver and natural compounds. Mater. Sci. Eng. C.

[23] Nguyen T.T.H., Si J., Kang C., Chung B., Chung D., Kim D. (2017). Facile preparation of water soluble curcuminoids extracted from turmeric (*Curcuma longa* L.) powder by using steviol glucosides. Food Chem..

[24] Kadota K., Okamoto D., Sato H., Onoue S., Otsu S., Tozuka Y. (2016). Hybridization of polyvinylpyrrolidone to a binary composite of curcumin/α-glucosyl stevia improves both oral absorption and photochemical stability of curcumin. Food Chem..

[25] Ruesgas-Ramón M., Figueroa-Espinoza M.C., Durand E. (2017). Application of deep eutectic solvents (DES) for phenolic compounds extraction: Overview, challenges, and opportunities. J. Agric. Food. Chem..

[26] Georgantzi C., Lioliou A.-E., Paterakis N., Makris D. (2017). Combination of lactic acid-based deep eutectic solvents (DES) with β-cyclodextrin: Performance screening using ultrasound-assisted extraction of polyphenols from selected native Greek medicinal plants. Agronomy.

[27] Almeida C.M.R., Magalhães J.M.C.S., Souza H.K.S., Gonçalves M.P. (2018). The role of choline chloride-based deep eutectic solvent and curcumin on chitosan films properties. Food Hydrocoll..

[28] Pereira P.F., Andrade C.T. (2017). Optimized pH-responsive film based on a eutectic mixture-plasticized chitosan. Carbohydr. Polym..

[29] Kroon L.P.N.M., Brouwer H., de Cock A.W.A.M., Govers F. (2012). The genus *Phytophthora* anno 2012. Phytopathology.

[30] Munnecke D.E. (1984). Establishment of micro-organisms in fumigated avocado soil to attempt to prevent reinvasion of the soils by *Phytophthora cinnamomi*. Trans. Br. Mycol. Soc..

[31] Zentmyer G.A., Erwin D.C., Bartnicki-García S., Tsao P.H. (1983). The world of Phytophthora. Phytophthora, Its biology, Taxonomy, Ecology and Pathology.

[32] Ali M., Kim B., Belfield K.D., Norman D., Brennan M., Ali G.S. (2015). Inhibition of *Phytophthora parasitica* and *P. capsici* by silver nanoparticles synthesized using aqueous extract of *Artemisia absinthium*. Phytopathology.

[33] Atia M.M.M., Buchenauer H., Aly A.Z., Abou-Zaid M.I. (2005). Antifungal activity of chitosan against *Phytophthora infestans* and activation of defence mechanisms in tomato to late blight. Biol. Agric. Hortic..

[34] Wang L.-S., Wang C.-Y., Yang C.-H., Hsieh C.-L., Chen S.-Y., Shen C.-Y., Wang J.-J., Huang K.-S. (2015). Synthesis and anti-fungal effect of silver nanoparticles-chitosan composite particles. Int. J. Nanomed..

[35] Silva-Castro I., Martín-García J., Diez J.J., Flores-Pacheco J.A., Martín-Gil J., Martín-Ramos P. (2017). Potential control of forest diseases by solutions of chitosan oligomers, propolis and nanosilver. Eur. J. Plant Pathol..

[36] Martín-Gil J., Matei Petruta M., Pérez Lebeña E. (2017). Complejo de inclusión para mejorar la biodisponibilidad de compuestos biológicamente activos no hidrosolubles.

[37] Kim M.K., Choi G.J., Lee H.S. (2003). Fungicidal property of *Curcuma longa* L. rhizome-derived curcumin against phytopathogenic fungi in a greenhouse. J. Agric. Food Chem..

[38] Pompimon W., Jomduang J., Prawat U., Mankhetkorn S. (2009). Anti-*Phytopthora capsici* activities and potential use as antifungal in agriculture of *Alpinia galanga* Swartz, *Curcuma longa* Linn, *Boesenbergia pandurata* Schut and *Chromolaena odorata*: Bioactivities guided isolation of active ingredients. Am. J. Agric. Biol. Sci..

[39] Hu L.-F., Chen C.-Z., Yi X.-H., Feng J.-T., Zhang X. (2008). Inhibition of p-isopropyl benzaldehyde and p-isopropyl benzoic acid extracted from *Cuminum cyminum* against plant pathogens. Acta Bot. Boreal. Occident. Sin..

[40] Shim S.-H., Kim J.-C., Jang K.-S., Choi G.-J. (2009). Anti-oomycete activity of furanocoumarins from seeds of *Psoralea corylifolia* against *Phytophthora infestans*. Plant Pathol. J..

[41] Krutyakov Y.A., Kudrinskiy A.A., Zherebin P.M., Yapryntsev A.D., Pobedinskaya M.A., Elansky S.N., Denisov A.N., Mikhaylov D.M., Lisichkin G.V. (2016). Tallow amphopolycarboxyglycinate-stabilized silver nanoparticles: New frontiers in development of plant protection products with a broad spectrum of action against phytopathogens. Mater. Res. Express.

[42] Banik S., Pérez-de-Luque A. (2017). In vitro effects of copper nanoparticles on plant pathogens, beneficial microbes and crop plants. Span. J. Agric. Res..

[43] Ostaszewska T., Chojnacki M., Kamaszewski M., Sawosz-Chwalibóg E. (2015). Histopathological effects of silver and copper nanoparticles on the epidermis, gills, and liver of Siberian sturgeon. Environ. Sci. Pollut. Res..

[44] Dobrochna A., Jerzy S., Teresa O., Magda F., Malgorzata R., Yuichiro M., Kacper M. (2018). Effect of copper and silver nanoparticles on trunk muscles in rainbow trout (*Oncorhynchus mykiss*, Walbaum, 1792). Turk. J. Fish. Aquat. Sci..

[45] Badawy M.E.I., Rabea E.I. (2016). Synthesis and antimicrobial activity of *N*-(6-carboxyl cyclohex-3-ene carbonyl) chitosan with different degrees of substitution. Int. J. Carbohydr. Chem..

[46] Kim K.-J., Sung W.S., Suh B.K., Moon S.-K., Choi J.-S., Kim J.G., Lee D.G. (2008). Antifungal activity and mode of action of silver nano-particles on *Candida albicans*. BioMetals.

[47] Clement J.L., Jarrett P.S. (1994). Antibacterial silver. Met. Based Drugs.

[48] Zhang J., Higashi K., Ueda K., Kadota K., Tozuka Y., Limwikrant W., Yamamoto K., Moribe K. (2014). Drug solubilization mechanism of α-glucosyl stevia by NMR spectroscopy. Int. J. Pharm..

[49] Ferreira F.D., Mossini S.A.G., Ferreira F.M.D., Arrotéia C.C., da Costa C.L., Nakamura C.V., Machinski Junior M. (2013). The inhibitory effects of *Curcuma longa* L. essential oil and curcumin on *Aspergillus flavus* Link growth and morphology. Sci. World J..

[50] Lee W., Lee D.G. (2014). An antifungal mechanism of curcumin lies in membrane-targeted action within *Candida albicans*. IUBMB Life.

[51] Badhani B., Sharma N., Kakkar R. (2015). Gallic acid: A versatile antioxidant with promising therapeutic and industrial applications. RSC Adv..

[52] Ing L.Y., Zin N.M., Sarwar A., Katas H. (2012). Antifungal activity of chitosan nanoparticles and correlation with their physical properties. Int. J. Biomater..

[53] Juneidi I., Hayyan M., Mohd Ali O. (2016). Toxicity profile of choline chloride-based deep eutectic solvents for fungi and *Cyprinus carpio* fish. Environ. Sci. Pollut. Res..

[54] Hayyan M., Hashim M.A., Al-Saadi M.A., Hayyan A., AlNashef I.M., Mirghani M.E.S. (2013). Assessment of cytotoxicity and toxicity for phosphonium-based deep eutectic solvents. Chemosphere.

[55] Radošević K., Čanak I., Panić M., Markov K., Bubalo M.C., Frece J., Srček V.G., Redovniković I.R. (2018). Antimicrobial, cytotoxic and antioxidative evaluation of natural deep eutectic solvents. Environ. Sci. Pollut. Res..

[56] Papaccio G., Hayyan M., Looi C.Y., Hayyan A., Wong W.F., Hashim M.A. (2015). In vitro and in vivo toxicity profiling of ammonium-based deep eutectic solvents. PLoS ONE.

[57] Mbous Y.P., Hayyan M., Wong W.F., Looi C.Y., Hashim M.A. (2017). Unraveling the cytotoxicity and metabolic pathways of binary natural deep eutectic solvent systems. Sci. Rep..

[58] Wikene K.O., Rukke H.V., Bruzell E., Tønnesen H.H. (2017). Investigation of the antimicrobial effect of natural deep eutectic solvents (NADES) as solvents in antimicrobial photodynamic therapy. J. Photochem. Photobiol. B Biol..

[59] Fan Y., Li X., Yan L., Li J., Hua S., Song L., Wang R., Sha S. (2017). Enhanced extraction of antioxidants from aqueous solutions by ionic liquids. Sep. Purif. Technol..

[60] Sun T., Zhou D., Xie J., Mao F. (2007). Preparation of chitosan oligomers and their antioxidant activity. Eur. Food Res. Technol..

[61] Biswas A., Shogren R.L., Stevenson D.G., Willett J.L., Bhowmik P.K. (2006). Ionic liquids as solvents for biopolymers: Acylation of starch and zein protein. Carbohydr. Polym..

[62] Raja S., Murty V.R., Thivaharan V., Rajasekar V., Ramesh V. (2012). Aqueous two phase systems for the recovery of biomolecules—A review. Sci. Technol..

[63] Şesan T.E., Enache E., Iacomi B.M., Oprea M., Oancea F., Iacomi C. (2017). In vitro antifungal activity of some plant extracts against *Fusarium oxysporum* in blackcurrant (*Ribes nigrum* L.). Acta Sci. Pol. Hortorum Cultus.

